# Association between local immune cell infiltration, mismatch repair status and systemic inflammatory response in colorectal cancer

**DOI:** 10.1186/s12967-020-02336-6

**Published:** 2020-04-21

**Authors:** Ulf Gunnarsson, Karin Strigård, Sofia Edin, Ioannis Gkekas, Harri Mustonen, Tuomas Kaprio, Camilla Böckelman, Jaana Hagström, Richard Palmqvist, Caj Haglund

**Affiliations:** 1 Department of Surgical and Perioperative Sciences, Umeå University, 901 87 Umeå, Sweden; 2 Department of Medical Biosciences/Pathology, Umeå University, 901 87 Umeå, Sweden; 3 Department of Surgery, University of Helsinki and Helsinki University Hospital, 00114 Helsinki, Finland; 4 Translational Cancer Medicine Research Program, Faculty of Medicine, University of Helsinki, 00014 Helsinki, Finland; 5 Department of Pathology, University of Helsinki and Helsinki University Hospital, 00014 Helsinki, Finland

**Keywords:** Colorectal cancer, Local immune response, Systematic inflammatory response, Miscrosatellite instability, Mismatch repair

## Abstract

**Background:**

Systemic inflammatory response in colorectal cancer (CRC) has been established as a prognostic factor for impaired cancer-specific survival, predominantly in patients with right-sided tumors. On the other hand, defective mismatch repair (dMMR) tumors, primarily located in the right colon, are known to have favorable survival and dense local immune infiltration. The aim of this study was to see if there is any form of relationship between these seemingly diverse entities.

**Methods:**

Complete clinical and long-term survival data were retrieved for 316 CRC patients operated at Helsinki University Hospital between the years 1998 and 2003. Tissue microarrays were prepared from surgical specimens and further processed and analyzed for local immune cell infiltration using multispectral imaging with a Vectra quantitative pathology imaging system and Inform software. Multiplex immunohistochemistry was applied using antibodies against CD66b, CD8, CD20, FoxP3, CD68 and pan-Cytokeratin. After exclusions, data on immune infiltration were available for 275 patients. Mismatch repair status was determined by immunohistochemistry.

**Results:**

CRP was seen to be an independent predictor of cancer-specific survival but not overall survival in uni- and multivariable (HR 1.01 (1.00–1.02); p = 0.028) analyses of non-irradiated patients. There was no significant difference in CRP according to mismatch repair status, but all cases (n = 10) with CRP ≥ 75 mg/l had proficient mismatch repair (pMMR). There was a significant negative correlation between intratumor stromal infiltration by T-regulatory FOXP3^+^ cells and CRP (p = 0.006). There was significantly lower intratumor stromal infiltration by FOXP3^+^ cells (p = 0.043) in the right colon compared to the rectum, but no significant difference in CRP (p = 0.44). CRP was not a predictor of overall survival (HR 0.99, 95% CI 0.98–1.01) nor cancer-specific survival in irradiated patients (HR 0.94, 95% CI 0.94–1.02).

**Conclusions:**

There was a significant negative relationship between SIR, defined as an elevated CRP, and intratumor stromal infiltration by T-regulatory FOXP3^+ ^cells. This and the fact that all cases with a CRP > 75 mg/l had pMMR suggests that SIR and dMMR are independent entities in CRC. Indeed, the general lack of difference in CRP between cases with dMMR and pMMR may be evidence of overlap in cases with a less pronounced SIR.

## Background

Systemic inflammatory response (SIR), defined as elevated circulating C-reactive protein (CRP), prior to colorectal cancer (CRC) surgery has been established as an independent risk factor for impaired survival [1, 2]. A subsequent study further verified the relationship between SIR and impaired survival for patients with CRC liver metastases operated with curative intent [3]. In that study no relationship was seen between size of metastases and survival, indicating that immunological mechanisms and SIR are the detrimental factors rather than tumor burden per se. SIR is thus a factor that increases the mortality of a form of cancer that is the third most deadly malignant disease worldwide [4]. In the first study by our group [1], impaired survival associated with SIR was revealed stage for stage, and high CRP alone was seen to be a stronger predictor than the Glasgow prognostic scale [5] combining high CRP and low serum albumin. However, elevation of CRP in CRC can be due to several interfering factors. It may well be that an elevated CRP resulting from an inflammatory response, anemia and hypoalbuminemia has broader prognostic significance, whereas elevated CRP due to a tumor-induced SIR is a strong prognostic factor in a well-defined population only [6].

Previous studies have shown that most SIR-inducing CRCs are right-sided [1, 7]. The right colon is commonly regarded as the most immunoreactive section of the colo-rectum [8, 9]. Tumors with defective mismatch repair (dMMR), which are also mainly located in the right colon, are seen to have strong local immune infiltration in the tumor microenvironment [10, 11]. Patients with dMMR tumors have a more favorable oncologic survival than patients with tumors that induce an SIR, where cancer-specific survival is impaired [1, 2, 12, 13]. Thus, two forms of CRC with divergent immune responses and cancer-specific survival are predominantly located in the right colon. Little is known, however, about relationships or interference between SIR-inducing tumors and dMMR tumors.

No study has been published describing the underlying immunologic mechanism behind SIR induction in CRC, or the mechanism by which SIR impairs oncologic survival in CRC. Increase in our knowledge of characteristics and local immune responses in SIR-inducing CRC tumors and their surroundings, is essential if we are to understand and possibly avert the detrimental processes associated with these tumors.

The aim of the present study was to compare local and systemic immune responses and MMR status with long-term outcome in a well-defined population of CRC patients, and how these interact to influence overall and cancer-specific survival.

The hypothesis of this study was that there is a relationship between tumor-induced SIR and tumors with dMMR in patients with right-sided CRC, and that there are common local tumor-associated immune events in these two entities.

## Methods

This study comprised 316 CRC patients operated on at Helsinki University Hospital between 1998 and 2003. Data were extracted from patient records and tissue samples from the pathology archives of the hospital. The local research register included data on date of diagnosis, gender, tumor location and stage of disease. CRP levels in preoperatively collected plasma samples, stored at -80 °C pending analysis, were obtained using a sensitive CRP assay at the local hospital laboratory [14]. Survival data and cause of death were gathered by matching the local research register with the Finnish Population Register and Statistics Finland. The mean and maximum follow-up times were 7.3 and 17.1 years respectively.

### Preparation for tissue microarrays

Marking of representative tumor areas was made on digitalized H&E slides by an experienced gastropathologist. The tissue microarray (TMA) blocks were generated by punching 1 mm cores from formalin-fixed and paraffin-embedded tissue samples using a TMA Grand Master 3D instrument (Histech Ltd Budapest, Hungary). Two 1 mm cores were taken from each tumor area, one central and one peripheral to avoid systematic heterogeneity.

### Multispectral imaging, analysis and data collection

Analysis of infiltrating immune cells using multiplexed immunohistochemistry and multispectral imaging has been described previously [15]. In brief, the TMA slides were sequentially stained with antibodies against CD66b, CD8, CD20, FoxP3, CD68 and pan-Cytokeratin. The VECTRA 3 Quantitative Pathology Imaging System (PerkinElmer) was used for multispectral imaging. All standard epi-flourescent filters were used; DAPI, FITC, CY3, Texas Red, and CY5. Whole slide scans were acquired using × 10 magnification. The Phenochart software (PerkinElmer) was subsequently used to mark TMA cores from the whole slide scans for subsequent multispectral imaging using x20 magnification. Images were quantified using the InForm software in two steps. Firstly, 15 TMA cores representing the heterogeneous nature of CRC, were selected to train machine-learning algorithms for tissue segmentation, cell segmentation and cell phenotyping. These were then applied to all TMA cores. Each scanned image was examined by one observer under the supervision of an experienced gastropathologist.

### Exclusion criteria and further processing of TMAs

TMA cores were excluded if one of the following criteria was present: large part or whole of core lost; tumor or stromal tissue missing; poor quality staining (e.g. pan-Cytokeratin stain too weak); or widespread necrosis. This resulted in final study samples from 275 patients. In 66 of these, infiltration of immune cells was evaluated in one TMA core only.

TMA cores were checked for irrelevant areas that were manually indicated and subtracted from the image. Such areas included: cellular debris; mucus; large vessels; areas of necrosis, large lymphoid aggregates and normal epithelial tissue. After this exclusion process, cell segmentation summary data were collected for each TMA core and converted to number of individual cell types per mm^2^ (tumor or stromal compartment). The average number of a particular cell per mm^2^ was calculated from the total number of cells divided by the total tissue area in cases where two TMA cores were included. Infiltrating immune cells, identified by the different markers, were further divided into groups of high and low infiltration by the median number of infiltrating cells.

### Determination of mismatch repair status

MMR screening status was determined by immunohistochemical analyses of TMA slides of the protein products of genes involved in the DNA mismatch repair system, as described previously [16]. Antibodies (all from Ventana Medical Systems Inc., Tucson, Arizona, US) against the four key mismatch repair proteins (MLH1 (clone M1), MSH2 (clone G219-1129), PMS2 (clone A16-4) and MSH6 (clone SP93)) were used on an automated Ventana BenchMark ULTRA instrument. Tumors with loss of nuclear expression of one or more of these proteins were described as dMMR, while samples with positive staining for all four antibodies were denoted pMMR. Tumors where internal staining was lacking, such as positive lymphocytes, were considered uninformative.

### Statistical methods

Statistica^®^ version 13 (StatSoft, Tulsa, OK, USA), STATA version 15.1 (STATACorp LLC, Texas, USA) and SPSS version 24 (IBM corp, Chicago, IL, USA) were used for statistical calculations. Non-parametric analyses were generally used. The Cox proportional hazard model was used for investigating associations between survival and predictor variables in uni- and multivariable analyses. CRP and age were included as continuous variables divided by 10. The assumption of constant hazard ratio over time was tested by including time-dependent variables for each variable in the model. Interactions were also considered in the multivariable models, but no significant interactions were found when correcting for multiple comparisons using the Bonferroni method. The multivariable models were adjusted for age, gender, stage and location of the tumor. In cancer specific survival analyses other deaths were taken as competing events and competing cox regression models were used. Kaplan–Meier curves were used to analyze disease specific and overall survival. Differences in outcome between groups were analyzed using log-rank test.

Comparison of continuous data between groups was made with the Mann–Whitney U test. Correlation between continuous variables was evaluated using the Spearman’s rank test and bias corrected and accelerated 95% confidence intervals for the Spearman’s rho were obtained by the bootstrap method (1000 replication). P values < 0.05 were considered statistically significant and two-tailed tests were used.

Irradiated patients were not included in the multivariable models. Possible effects of SIR were evaluated separately in this group.

### Ethics

Ethics approval was given by the Surgical Ethics Committee of Helsinki University Hospital (Dnro HUS 226/E6/06, extension TMK02 §66 17.4.2013). The use of tissue specimens was approved by The National Supervisory Authority of Health and Welfare (Valvira Dnro 10041/06).

## Results

In all, 275 cases were included in the study. Basic characteristics of the patients are shown in Table 1. Patients had a mean age of 67.9 years and distribution of gender was similar.

**Table 1:** Basic patient characteristics

Age years, mean (SD)	67.9 (12.2)
Female gender	132 (48%)
Stage I	55 (20%)
Stage II	77 (28%)
Stage III	95 (35%)
Stage IV	48 (17%)
Right colon	73 (27%)
Left colon	55 (20%)
Rectum non-irradiated	99 (36%)
Rectum irradiated	48 (17%)

Basic patient characteristic for the 275 patients included. Data are n (%) unless otherwise stated

We found that CRP was a significant predictor of cancer-specific survival (Table 2a) in both the uni- and multivariable models but for overall survival (Table 2b) in the multivariable model only, after exclusion of irradiated patients. Localization of the tumor was not a predictor of overall nor cancer-specific survival in the univariable model. Age and stage were predictors of survival in both models. Overall and cancer specific survival according to CRP and MMR status, stratified by infiltration of FOXP3^+^ cells, are illustrated in Kaplan–Meier curves (Additional file 1 and Additional file 2).

**Table 2:** Uni- and multivariable analyses of (**a**) cancer-specific and (**b**) overall survival in non-irradiated CRCs

Variable	Univariable		Multivariable	
	HR (95%CI)	*p*	HR (95%CI)	*p*
(a) Cancer survival^a^				
Female gender	1.17 (0.74–1.83)	0.503		
Right colon	1.00 (Reference)		1.00 (Reference)	
Left colon	1.45 (0.77–2.71)	0.251	1.14 (0.60–2.17)	0.682
Rectum	1.54 (0.88–2.70)	0.128	1.04 (0.58–1.89)	0.888
Stage I	0.09 (0.02–0.37)	0.001	0.08 (0.02–0.33)	< 0.001
Stage II	0.34 (0.17–0.68)	0.002	0.25 (0.12–0.51)	< 0.001
Stage III	1.00 (Reference)		1.00 (Reference)	
Stage IV	3.08 (1.91–4.97)	<0.001	3.43 (2.02–5.84)	< 0.001
CRP (per 10mg/l)	1.09 (1.01–1.17)	0.022	1.08 (1.00–1.15)	0.042
Age (per 10 years)	1.24 (1.02–1.51)	0.032	1.59 (1.30–1.95)	< 0.001
(b) Overall survival^b^				
Female gender	1.10 (0.78–1.54)	0.60		
Right colon	1.00 (Reference)		1.00 (Reference)	
Left colon	0.79 (0.49–1.28)	0.49	0.87 (0.52–1.44)	0.58
Rectum	1.18 (0.80–1.73)	0.79	1.10 (0.73–1.64)	0.66
Stage I	0.48 (0.28–0.81)	0.006	0.40 (0.23–0.70)	0.001
Stage II	0.71 (0.46–1.10)	0.12	0.50 (0.32–0.80)	0.003
Stage III	1.00 (Reference)		1.00 (Reference)	Reference
Stage IV	2.40 (1.53–3.76)	< 0.001	3.13 (1.93–5.06)	< 0.001
CRP (per 10mg/l)	1.05 (0.99–1.12)	0.096	1.08 (1.01–1.15)	0.037
Age (per 10 years)	2.00 (1.68–2.38)	< 0.001	1.66 (1.25–2.20)	< 0.001

^a^CRP and age were included as continuous variables in steps of 10 units. The multivariable analysis was stratified for gender. Other deaths used as competing events

^b^CRP and age were included as continuous variables in steps of 10 units divided by 10. The multivariable analysis was stratified for gender and a time-dependent correction term for age (1.10/year, 95% CI 1.03–1.17, *p*=0.004) was added to account for deviation from the Cox model assumption of constant hazard ratio over time

Among irradiated rectal cancer patients, CRP (divided by ten) level was not a predictor of overall (HR 0.95, 95% CI 0.83–1.08) nor cancer-specific survival (HR 0.83, 95% CI 0.55–1.25).

Of the 275 cases analyzed for local infiltration of immune cells, it was possible to determine MMR status in 248 cases. Of these, 221 were pMMR and 27 (10.9%) dMMR tumors. When restricting the analysis to non-irradiated patients as in the multivariable analyses, the corresponding figures were 183 and 26 (12.4%). There was no significant difference in CRP levels between dMMR and pMMR cases (median 4.9, IQR 1.7–15.6 and median 3.1, IQR 1.7–16.4 for pMMR and dMMR, respectively, *p *= 0.785). However, all cases (n = 10) with a CRP > 75 g/l were pMMR. Neither when restricting analyses to the right colon, CRP level (median 6.6, IQR 1.7–17.2 and median 3.0, IQR 1.6–17.4 for pMMR and dMMR, respectively, *p *= 0.693) was related to MMR status.

Infiltration of the tumor stroma by T-regulatory FOXP3^+^ cells showed a significantly negative low correlation to CRP (Spearman’s rho –0.18, 95% CI from –0.32 to –0.05 for all non-irradiated and –0.24, 95% CI from –0.42 to –0.05 for non-irradiated rectal cancers (Table 3).

**Table 3:** Correlation between CRP and immune cell infiltration

	*N*	Stroma CD20	Stroma CD66b	Stroma CD68	Stroma CD8	Stroma FOXP3	Tumor CD66b	Tumor CD8
All non-irradiated	227	−0.042 (*p *= 0.52)	−0.085 (*p *= 0.20)	−0.106 (*p *= 0.11)	−0.013 (*p *= 0.84)	−0.18 (*p *= 0.006)	−0.087 (*p *= 0.19)	−0.018 (*p *= 0.78)
Rectum non-irradiated	99	−0.141 (*p *= 0.16)	−0.131 (*p *= 0.20)	−0.121 (*p *= 0.23)	−0.001 (*p *= 0.99)	−0.235 (*p *= 0.019)	−0.144 (*p *= 0.16)	−0.051 (*p *= 0.62)
Rectum irradiated	48	0.116 (*p *= 0.43)	−0.134 (*p *= 0.36)	−0.275 (*p *= 0.058)	−0.090 (*p *= 0.54)	−0.163 (*p *= 0.27)	0.137 (*p *= 0.35)	−0.093 (*p *= 0.53)

Spearman rank correlation between preoperative CRP and local infiltration by specific immune cell types (average cells/mm^2^)

There was a negative but not significant relationship between CRP and tumor epithelial and tumor stromal infiltration of all other immune cells analyzed by the Vectra palette (Table 3). The correlation between CRP and infiltration of the intratumor stroma by T-regulatory FOXP3^+^ cells was more pronounced in dMMR than pMMR cases (–0.53, 95% CI from –0.79 to –0.12; *p *= 0.006 and –0.18, 95% CI from –0.32 to –0.017; *p *= 0.014 respectively for non-irradiated patients). There was no significant correlation between dMMR and pMMR cases for any of the other infiltrating immune cells assessed (data not shown).

**Fig. 1:**
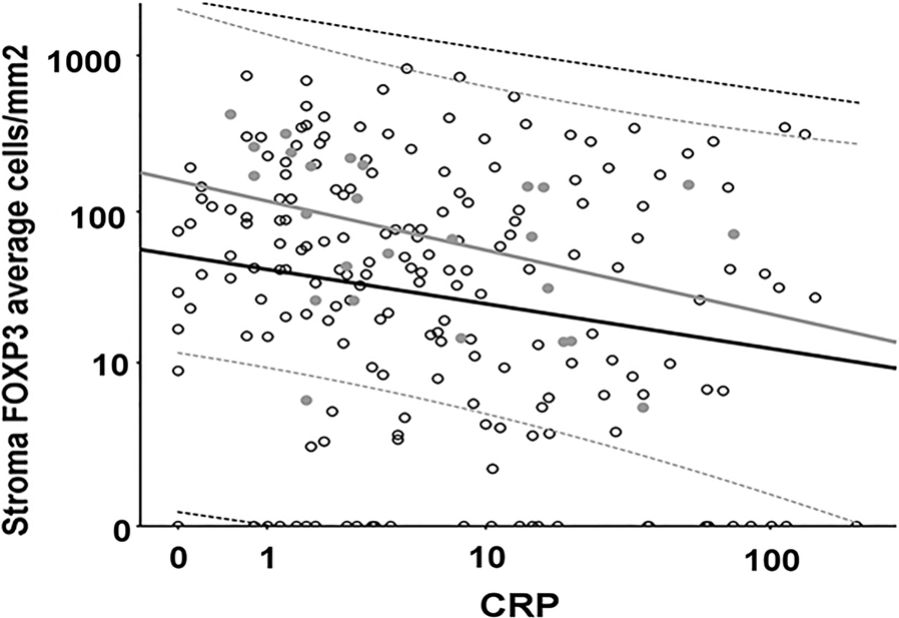
Scatterplot of tumor stroma infiltration of FOXP3^+^ T-regulatory cells versus CRP Infiltration T-regulatory FOXP3^+ ^immune cells in the tumor stroma (cells/mm2) plotted against CRP (mg/l) for pMMR (open circles) and dMMR (grey dots) patients. Linear regression lines with 95% CI plotted for pMMR (black lines) and dMMR (grey lines)

It should be mentioned that among non-irradiated patients there were several cases with a very low CRP level and low infiltration of FOXP3^+^ cells in the intratumor stroma despite the significant negative correlation (Fig. 1). Infiltration of intratumoral T-regulatory FOXP3^+^ cells in the stroma correlated with local infiltration of all other investigated immune cells; *p *= 0.002 with stromal CD66b cells and *p *< 0.001 with the other immune cells.

In non-irradiated patients, intratumor stromal FOXP3^+^ cell infiltration was greater in the right than in the left colon/rectum (67.5, IQR 14.3–197.4 and 29.1, IQR 5.9–88.5 (cells/mm2) respectively, p = 0.044).

## Discussion

An inverse relationship between systemic inflammatory response (defined as elevated CRP) and infiltration of T-regulatory FOXP3^+^ immune cells in the intratumor stroma was seen in this well-defined cohort of CRC patients with long-term follow-up. To our knowledge, this is the first time local immune response in the tumor microenvironment has been compared to SIR in CRC. An inverse relationship was also seen with other immune cells but to a lower degree. The fact (Fig. 1) that there were several cases with low CRP and low FOXP3^+^ cell infiltration indicate heterogeneity which may be dependent on a third factor. This is congruent with the hypothesized overlap between cases with dMMR and SIR, and this will be evaluated in further targeted studies.

In CRC patients, a high infiltration of FOXP3^+^ cells has been linked to an improved prognosis [17, 18], which is in contrast to what has been shown for many other cancer types *e.g.* renal cancer and breast cancer [19]. Furthermore, over the past decade SIR has been established as a strong negative prognostic factor [1, 2], which was also the case in the present study. The negative correlation between tumor stromal infiltration by FOXP3^+^ lymphocytes and SIR defined as elevated plasma CRP, as described in the present study, is congruent with that observation. However, a positive correlation between CRP level and FOXP3^+^ cell tumor infiltration has been observed in clear cell renal cell carcinoma (RCC) [20]. The biological explanation for the suggested inverse relationship between CRP and FOXP3^+^ cells in CRC remains speculative, and calls for further investigations. However, the strong positive correlation of FOXP3^+^ cells to other infiltrating immune cells, including CD8^+^ cytotoxic T cells, suggests that SIR is not likely to be the result of a strong local anti-tumor immune response in CRC. This emphasizes the fundamental differences that exist between different forms of cancer, and that the immune response may vary depending on both tumor- and organ-specific factors. Mechanisms by which the FOXP3 complex regulates infiltration of the tumor microenvironment by other immune cells at the molecular level are still not fully understood. FOXP3^+^ T-regulatory cells are considered to play a key role in balancing different immune mechanisms and in maintaining immune homeostasis. It is thought that expression of specific genes interacts with the FOXP3 protein in T cells. For example, removal of the DBC1 gene in a breast cancer mouse model increased resistance to experimental autoimmune manifestations by attenuating FOXP3 degradation [20]. The present data do not identify any single precipitating factor for SIR in CRC but indicate that infiltration of FOXP3^+^ T-regulatory cells may have an effect on cancer-specific survival.

In the present study, we found a fall in degree of infiltration of T-regulatory FOXP3^+^ immune cells in the intratumor stroma of cancers from the right- to the left colon/rectum (excluding cases of irradiated rectum cancer). To our best knowledge this observation has not been published previously, but it is in accordance with the results of several tumor marker studies [8, 9], and with a recent study evaluating local lymphocyte infiltration in H&E slides [7]. The latter study [7] also found a corresponding fall in SIR that was not reproduced in the present study. Other studies have also described differences in the occurrence of SIR between cancers of the right and left colon [1, 2].

The proportion of dMMR cases in the present study was relatively small, and probably the result of a higher proportion of rectal cancers included due to the healthcare structure in the Helsinki region.

In contrast to the results of two studies [22, 23], CRP was not an independent predictor of survival among irradiated rectal cancer patients in this study. One reason for this may be that both previous studies analyzed patients after combined chemo-radiotherapy, while irradiated patients in the present study comprised patients receiving combined therapy and patients receiving short-term radiotherapy immediately followed by surgery. It may also be that the timing of CRP sampling some weeks after chemo-radiotherapy reflects recovery and reorganization of the immune system in response to the chemotherapy, as described in cases of urine bladder cancer [24], which may potentiate its antitumoral effect.

No difference in CRP levels was seen between dMMR and pMMR tumors which supports our hypothesis that there is a relationship between these two entities. The observation that all cases with a CRP > 75 mg/l were pMMR tumors, however, suggests that cases with the most pronounced SIR were predominantly those with a systemic and not a local immune response. This agrees with the observed negative correlation between infiltration of FOXP3^+^ T-regulatory cells in the intratumor stroma and CRP level (Table 3). The fact (Fig. 1) that there were several cases with low CRP and low FOXP3^+^ cell infiltration indicate heterogeneity which may be dependent on a third factor. Interestingly this negative correlation was even more pronounced among dMMR cases, suggesting overlap and variation in the mix of immune responses underlying the homeostatic role of FOXP3 [21]. Furthermore, there was a significant positive correlation between tumor stromal infiltration by FOXP3^+^ T-regulatory cells and local infiltration of the other immune cell types investigated. Thus, a lower degree of FOXP3^+^cell infiltration was associated with a lower degree of infiltration of CD8^+^ cytotoxic T-cells, although infiltration by CD8^+^ cells was not statistically correlated to CRP.

When interpreting the regression plot in Fig. 1, all cases with a CRP higher than 75 mg/l were pMMR, whereas cases with lower CRP levels included both dMMR and pMMR tumors and no statistically significant difference was seen. The distribution of MMR status also served as a measurement of internal validity, since it is unlikely that other reasons for elevated CRP, such as autoimmune disease or infection, were missed when scrutinizing the records since all cases with the highest CRP had the same MMR status (pMMR).

As a measurement of external validity in the present study, CRP was verified as a predictor of cancer-specific survival but not of overall survival (Table 2a, b), which is in accordance with previously published data from our group [1] and the previously mentioned meta-analysis [2]. Several factors influence overall survival after surgery for CRC such as comorbidity and factors associated with higher age. Thus, the impact of SIR on cancer-specific survival observed in the present study speaks in favor of a true effect on the patient’s tumor characteristics. When analyzing a non-characterized crude population, other reasons for elevated CRP may attenuate the impact of SIR on the oncologic prognosis [6]. For this reason, the impact of SIR on survival seen in the present multivariable analysis necessitates a carefully controlled study population where reasons for elevated CRP levels other than SIR have been excluded.

A weakness of the present study is that it does not include cases where surgery was performed as an emergency. A previous study showed impaired oncologic survival stage for stage in this group of patients but was unable to explain the reason for this [25]. It was hypothesized that one reason could be differences in healthcare structure and emergency management, but a further study failed to verify this [26]. We suggest that immune mechanisms yet to be determined, both local immune infiltration and systemic immune responses to the tumor may well be key factors behind this phenomenon.

Another weakness is the use of TMAs and not whole tissue sections for the evaluation of local immune infiltration. The multispectral Vectra technique work flow is primarily designed for TMAs and not for whole tissue sections. To minimize the risk of influences from systematic intratumoral heterogeneity, tumour immune infiltration was evaluated from two individual TMA cores from within the tumor mass, one more central and one more peripheral. The novel multispectral Vectra technique adds complex information of local immune cell infiltration and the relation to SIR, however, the findings need to be validated in future studies.

## Conclusions

In conclusion there was a significant negative relationship between SIR defined as an elevated CRP and infiltration of T-regulatory FOXP3^+^ cells in the intratumor stroma. This, and the fact that all cases with a CRP > 75 mg/l were pMMR tumors suggests that SIR and dMMR are two separate entities. On the other hand, the absence of an overall difference in CRP between dMMR and pMMR tumors favors an overlap in cases with less pronounced SIR. Thus, our hypothesis of a partial overlap holds true when restricted to cases with lower degrees of SIR.

## Supplementary information

**Additional file 1: Figure S1.** Kaplan-Meier curves for disease specific (DSS) and overall survival (OS) for pMMR and dMMR cases separately, comparing CRP levels among different levels of infiltration by T-regulatory FOXP3^+^ immune cells in the tumoral stroma (cells/mm2). Median was used as a cut-off value for infiltration of FOXP3^+^ cells, and the 90th percentile for CRP. There were no disease specific events in dMMR cases with FOXP3^+^ ≤42. p-values are for comparison between different levels of CRP.

**Additional file 2: Figure S2.** Kaplan-Meier curves for disease specific (DSS) and overall survival (OS) for pMMR and dMMR cases separately, comparing CRP levels among different levels of infiltration by T-regulatory FOXP3^+^ immune cells in the tumoral stroma (cells/mm2). Median was used as a cut-off value for infiltration of FOXP3^+^ cells, and the 90th percentile for CRP. There were no disease specific events in dMMR cases with FOXP3^+^ ≤ 42. p-values are for comparison between different levels of CRP.

## Data Availability

The datasets analyzed during the current study are not publicly available due to Swedish and Finnish legislation, but anonymized data are available from the corresponding author on reasonable request.

## Abbreviations

CRC: Colorectal cancer
CRP: C-reactive protein
dMMR: Defective mismatch repair
MMR: Mismatch repair
pMMR: Proficient mismatch repair
SIR: Systemic inflammatory response
TMA: Tissue microarray
