# Supplementary material for: Association between local immune cell infiltration, mismatch repair status and systemic inflammatory response in colorectal cancer
Source: J Transl Med. 2020 Apr 21;18:178. doi: 10.1186/s12967-020-02336-6 (PMC7175507; doi:10.1186/s12967-020-02336-6)
Supplement: Supplementary file 2 — Additional file 2: Figure S2. Kaplan-Meier curves for disease specific (DSS) and overall survival (OS) for pMMR and dMMR cases separately, comparing CRP levels among different levels of infiltration by T-regulatory FOXP3+ immune cells in the tumoral stroma (cells/mm2). Median was used as a cut-off value for infiltration of FOXP3+ cells, and the 90th percentile for CRP. There were no disease specific events in dMMR cases with FOXP3+ ≤ 42. p-values are for comparison between different levels of CRP. [file 12967_2020_2336_MOESM2_ESM.pptx]

## Slide 1
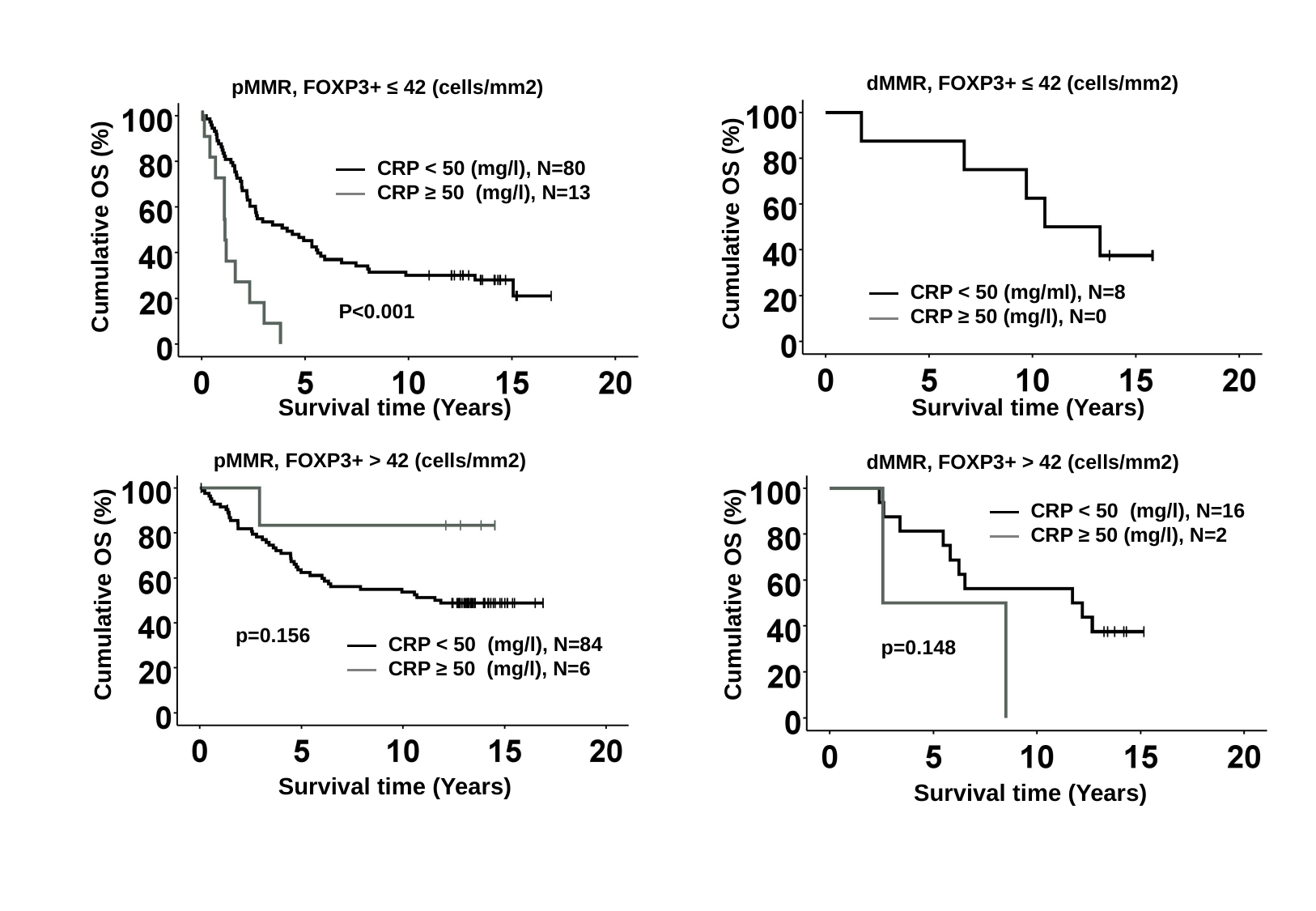

dMMR, FOXP3+ ≤ 42 (cells/mm2)
pMMR, FOXP3+ ≤ 42 (cells/mm2)
CRP < 50 (mg/l), N=80
CRP ≥ 50 (mg/l), N=13
Cumulative OS (%)
Cumulative OS (%)
CRP < 50 (mg/ml), N=8
CRP ≥ 50 (mg/l), N=0
P<0.001
Survival time (Years)
Survival time (Years)
pMMR, FOXP3+ > 42 (cells/mm2)
dMMR, FOXP3+ > 42 (cells/mm2)
CRP < 50 (mg/l), N=16
CRP ≥ 50 (mg/l), N=2
Cumulative OS (%)
Cumulative OS (%)
p=0.156
CRP < 50 (mg/l), N=84
CRP ≥ 50 (mg/l), N=6
p=0.148
Survival time (Years)
Survival time (Years)
